# Feasibility of therapeutic plasma exchange-based combination therapy in the treatment of acquired hemophilia A: A retrospective 6 case series

**DOI:** 10.1097/MD.0000000000026587

**Published:** 2021-07-23

**Authors:** Lin-Yue Wang, Yan Shen, Han-Qing Zeng, Ying Zhang, Shi-Feng Lou, Jian-Chuan Deng, Yun Luo

**Affiliations:** Department of Hematology, The Second Affiliated Hospital, Chongqing Medical University, Chongqing, China.

**Keywords:** combined modality therapy, corticosteroids, dexamethasone, factor VIII deficiency, hemophilia A, immunosuppressive agents, methylprednisolone, multimodal treatment, plasma exchange, rituximab

## Abstract

Poor availability and a lack of affordability of bypassing agents (recombinant activated factor VII and activated prothrombin complex concentrate) in west China prompted us to investigate an alternative cost-effective combination therapy. We aimed to explore the feasibility of therapeutic plasma exchange (TPE)-based combination therapy in the treatment of acquired hemophilia A (AHA).

We retrospectively investigated the clinical features of AHA in 6 patients who were treated with a combination of TPE, corticosteroids, and rituximab in our department for 9 years between January, 2011 and December, 2019.

We examined 1 male and 5 female patients. The median age at diagnosis of AHA was 51 years (18–66 years). In all patients, FVIII activity levels were low (median: 1.5%; 1–3%), FVIII inhibitor titers were high (median: 24.5 BU/mL; 13.2–48.6 BU/mL), and activated partial thromboplastin time was markedly prolonged (median: 99.4 s; 60.9–110.1 s). They underwent 2 to 8 cycles of plasma exchange and were given varying combinations of dexamethasone, methylprednisolone, prednisone, and rituximab. After TPE bleeding gradually stopped, and activated partial thromboplastin time decreased. After 3 months of treatment, FVIII inhibitors completely disappeared.

TPE when combined with corticosteroids and rituximab, as adjunctive immunosuppressive agents, may be an effective and reliable treatment for AHA. When there is no alternative, intensive first-line treatment including TPE may be lifesaving.

## 1 Introduction

Acquired hemophilia A (AHA), an autoimmune disorder characterized by antibody-mediated depletion of coagulation factor VIII (factor VIII, FVIII), may cause spontaneous or severe hemorrhage.^[1]^ Auto-antibodies directed against epitopes C2 or A2 and A3 of FVIII define this rare disorder with an estimated incidence of 1.2 to 1.5 cases per million individuals per year.^[2]^ This disease is mostly diagnosed in elderly individuals, aged 65 years or older with a preponderance in women of childbearing age,^[2]^ where an increased risk of approximately 8.4% has been reported.^[1]^ Although in about 51.9% of cases the disease remains unexplained or “idiopathic,”^[3]^ AHA coexists with neoplasm (11.8%), autoimmune diseases (11.6%), such as rheumatoid arthritis, systemic lupus erythematosus (SLE), and autoimmune thyroid disease, Sjögren syndrome, antiphospholipid syndrome, and other connective tissue diseases. Further, it has been shown to be triggered by infections (3.8%), induction by drugs (3.4%), monoclonal gammopathy of undetermined significance (2.6%), polymyalgia rheumatica (2.2%), dermatological diseases (1.4%), and reaction after transfusion of blood products (0.8%).^[1]^

Two basic management strategies for patients with AHA include hemostatic therapy to control active bleeding episodes and immunosuppressive therapy to reduce further risk of bleeding by decreasing the levels of anti-FVIII autoantibody inhibitor. Consequently, severe and potentially life-threatening bleeding episodes often warrant immediate hemostatic therapy. According to an international expert panel from the Hemostasis and Thrombosis Research Society of North America, recombinant factor VIIa (rFVIIa, NovoSeven) and activated prothrombin complex concentrate (aPCC, FEIBA-plasma-derived, containing factors II, IX, X, and VIIa) have been endorsed as appropriate first-line bypassing treatments. Further, for patients in geographic locations where other hemostatic alternatives are not readily available, recombinant porcine FVIII was shown to be a self-sufficient hFVIII replacement therapy, albeit, its administration could trigger an increase in the anti- recombinant porcine FVIII titer in some patients^[4]^ and subsequently reduce the treatment efficacy.^[5]^ Most published protocols and treatment regimens recommend autoantibody eradication as soon as the diagnosis is made. Several approaches to eradicate inhibitors in patients who fail to respond to bypass therapy include, the use of immune-suppressive medications,^[6]^ substitution of FVIII by intravenous immunoglobulin,^[7]^ plasmapheresis,^[8]^ and immunoadsorption.^[9–12]^

Interestingly, the role of plasma exchange in eradicating autoantibodies of FVIII was explored as early as 1969.^[13]^ Further, Zeitler et al reported that the Bonn-Malmo protocol achieved rapid bleeding cessation with an undetectable inhibitor at a median of 3 days and complete remission in 88% of patients with a median of 14 days. These protocols, however, involved a combination of immunoadsorption, therapeutic plasma exchange (TPE), intravenous immunoglobulin, immunosuppression with cyclophosphamide and corticosteroids, and FVIII administration.^[12]^ The rationale for using TPE in AHA is the same as its use in thrombotic thrombocytopenic purpura (TTP). In TTP, TPE is used to quickly remove the inhibitor, supply protease enzymes (ADAMSTS), and then these patients can achieve remission with rituximab and immunosuppressants. Moreover, a recent case report demonstrated that TPE resulted in complete recovery of life-threatening bleeding in a patient with AHA who was non-responsive to immunosuppressant therapy and had high titers of coagulation inhibitors.^[14]^ Nevertheless, the role of plasma exchange in the treatment of AHA is unclear. Moreover, the first-line rFVIIa or aPCC treatments (hemostatic bypassing agents) for AHA are either, not easily available, or unaffordable for patients in third world countries, especially in West China.

Therefore, in view of the above and due to the paucity of literature on TPE in the treatment of AHA, in this retrospective case series study, 6 patients who were treated with a combination of TPE, corticosteroids, and rituximab in our department for 9 years between January, 2011 and December, 2019 were examined and their clinical features were analyzed. Further, we determined the feasibility of TPE-based combination therapy including, adjunctive immunosuppressive agents (corticosteroids and rituximab) in the effective treatment of AHA.

## 2 Methods

This study was approved by the Ethics committee of the Second Affiliated Hospital of Chongqing Medical University, Chongqing, China. In this retrospective case series, 6 patients with a confirmed diagnosis of AHA admitted to the Second Affiliated Hospital of Chongqing Medical University between January, 2011 and December, 2019 were examined. All patients consulted with our department for hemorrhage of unknown cause accompanied by anemia and prolonged activated partial thromboplastin time (aPTT). Their clinical data including, age, sex, hemorrhagic sites, aPTT value (seconds), hemoglobin (Hb) (g/dL), and the amount and the presence or absence of red blood cell (RBC) transfusions on admission were reviewed.

Hemorrhage was defined as the progression to anemia with less than 7.0 g/dL of Hb requiring RBC transfusion, or severe, life-threatening organ bleeding. RBC transfusions were performed using blood from unrelated donors (allogeneic) to avoid the risk of exposure to multiple donors. FVIII activity levels (%), inhibitor titers (Bethesda units; BU/mL), immunosuppressive drugs, and changes in FVIII inhibitors and the associations between the FVIII activity levels/inhibitor titers and the severity of hemorrhage were investigated. Coagulation factor assays were performed using a one-stage clotting assay to measure FVIII clotting activity.^[15]^ The classical Bethesda-based FVIII inhibitor assay was performed by following a 2-hour incubation period at 37°C, with a set cutoff value of <0.5 BU/mL, to determine the residual FVIII activity.^[16,17]^ In brief, the presence or absence of an FVIII inhibitor can be determined by comparing the differences in FVIII activity between the incubation mixture and a control mixture. High IgG antibody titers were defined as titers of >5 BU and low IgG antibody titers were defined as titers of <5 BU, according to the International Society of Hemostasis and Thrombosis. FVIII autoantibodies are polyclonal IgG antibodies that belong to IgG1 and IgG4 subclasses.^[18]^

Plasma exchange was performed using a continuous-flow centrifugal apheresis system (SPO, The Spectra Optia, Terumo BCT, Tokyo, Japan) with fresh frozen plasma as replacement fluid for rapid removal of circulating autoantibodies. Subsequently, we administered prednisolone at a dose of 1 mg/kg/d orally from day 1 until remission (dose reduction) following a low-dose infusion of rituximab (Roche Pharmaceutical Co., Ltd., Shanghai, China) at a fixed dose of 100 mg. For this study, we defined complete remission (CR) as normal FVIII activity (70–140%) and undetectable FVIII inhibitor titer levels, and partial remission (PR) as restored FVIII coagulant activity that is >30%, reduction of FVIII inhibitor titer levels to <5 BU/mL, and the absence of bleeding that is required hemostatic treatments.^[11]^

### 2.1 Statistical analyses

All data were analyzed using SPSS software (v23.0, IBM Corp., Armonk, NY, USA). Data are represented as median with interquartile range.

## 3 Results

### 3.1 Patient characteristics and bleeding sites

Table 1 summarizes the clinical characteristics of 6 patients with AHA. There was 1 male and 5 female patients. Among them, 3 patients had autoimmune diseases including, undifferentiated connective tissue disease, Sjogren syndrome, and SLE. One of the 5 female patients presented with AHA after delivering a full-term baby. The distribution of patient age and other coagulation profile factors are shown in Table 1. Ages at diagnosis of AHA were 18 to 66 years (median age: 51 years). FVIII activity levels were severely low (median:1.5%; 1%–3%), FVIII inhibitor titers were high (median: 24.5 BU/mL; 13.2–48.6 BU/mL), and aPTT values were markedly prolonged (median: 99.4 s; 60.9–110.1 s). All patients with AHA displayed high titers of autoantibodies.

**Table 1:** Summary of clinical characteristics of patients with AHA.

Case.	Age (years)	Gender	Underlying diseases	Site of hemorrhage	Hemoglobin levels (g/dL)	aPTT (seconds)	FVIII activity levels (%)	Inhibitor titers (BU/mL)	Number of cycles of TPE	Follow-up duration (months) and outcome
Case #1	66	Female	Suspected MDS	Hematoma of lower limbs	5.8	106.5	1	16.4	2	104 months, surviving (no relapse)
Case #2	53	Female	None	Hematoma of lower limbs, gingival bleeding	6.2	60.9	3	13.2	2	30 months, surviving (no relapse)
Case #3	49	Female	Undifferentiated connective tissue disease	Hematoma of lower limbs	7.1	80.7	2	48.6	2	11 months, relapsed and died of ARDS
Case #4	33	Female	None	Vaginal bleeding due to delivery	8.4	99.9	1	22.8	5	24 months, surviving (no relapse)
Case #5	64	Male	Sjogren syndrome	Retroperitoneal hematoma of lower limbs	6.2	98.8	1	26.2	3	18 months, surviving (no relapse)
Case #6	18	Female	SLE	Gastrointestinal hemorrhage	6.2	110.1	1	34.6	8	1 month, died of MOFS
Total	51 (18–66)	–	–	–	–	99.4 (60.9–11)	–	24.5 (13.2–48.6)	–	–

AHA = acquired hemophilia A, aPTT = activated partial thromboplastin time, ARDS = acute respiratory distress syndrome, BU = Bethesda units (high, if the value is >5 BU), MDS = myelodysplastic syndrome, MOFS = multiple organ failure syndrome, SLE = systemic lupus erythematosus, TPE = therapeutic plasma exchange.

**Figure 1:**
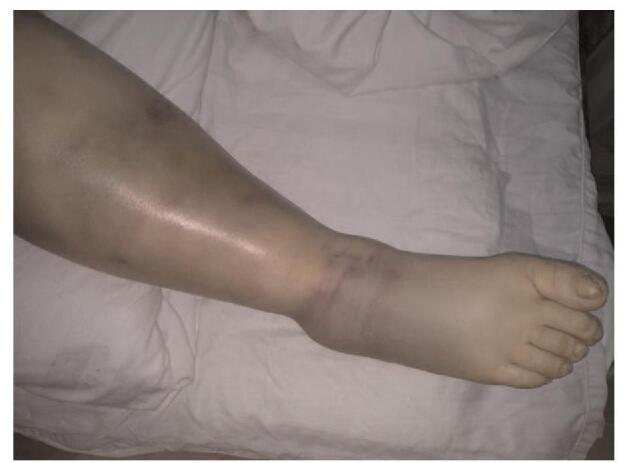
Subcutaneous hematoma in the right lower limb (case 3).

Further, lower extremity hemorrhages were common hemorrhagic sites (Fig. 1). Four out of 6 patients showed subcutaneous bleeding of their lower limbs, 1 of them had vaginal bleeding, and another had a gastrointestinal hemorrhage.

### 3.2 TPE and immunosuppressant drug dosing regimens

Table 2 summarizes the dosing regimen of TPE and immunosuppressant drugs.

**Table 2:** Dosing regimen of therapeutic plasma exchange and immunosuppressant drugs in AHA treatment.

Case	Hemorrhage site	Treatment regimen and dosage	Day to admission for TPE	Duration of therapy (days)	Remission
Case #1	Hematoma of lower limbs	PE + DEX + PON + R	11, 12	50	Partial
Case #2	Hematoma of lower limbs, gingival bleeding	PE + DEX + PON + R	2, 3	70	Complete
Case #3	Hematoma of lower limbs	PE + MP + PON + R	2, 3	16	Partial
Case #4	Vaginal bleeding due to delivery	PE + DEX + PON + R	2, 3, 16, 17, 18	75	Complete
Case #5	Retroperitoneal hematoma of lower limbs	PE + DEX + PON + R	6, 9, 11	13	Partial
Case #6	Gastrointestinal hemorrhage	PE + MP	13, 14, 15, 16, 18, 20, 22, 24	25	No

DEX = dexamethasone, MP = methylprednisolone, PE = plasma exchange, PON = prednisone, R = rituximab.

Individualized treatments were based on age, underlying disorders, bleeding site and severity, and inhibitor levels.^[19,20]^

All 6 patients were given varying combinations of corticosteroids (dexamethasone, methylprednisolone, and prednisone) and rituximab. All patients had undergone 2 to 8 cycles of plasma exchange. After plasma exchange, bleeding symptoms improved and aPTT decreased. After 3 months of treatment, FVIII inhibitors completely disappeared in all patients. Follow-up for 44 months (18–104 months) showed no recurrence of hemorrhage and FVIII inhibitors in 4 patients. Except for 2 patients, the others went on to experience remission after the maintenance therapy was stopped.

There were no adverse events identified that were related to TPE in any of the 6 cases.

### 3.3 Case studies

**Case #1:** Diagnosed with hematoma of lower limbs with suspicion of a pre-existing myelodysplastic syndrome (MDS) at another hospital. Her initial aPTT was 106.5 s and FVIII inhibitor titers were 16.4 BU/mL. She was given a combination of corticosteroids (dexamethasone 20 mg/d for 4 days and then this was changed to prednisone 1 mg/kg/d orally) as induction therapy and 2 cycles of plasma exchange were performed on days 11 and 12 of admission. Subsequently, there were no bleeding symptoms and aPTT markedly decreased. A full evaluation of the patient details suggested that the diagnosis of MDS was incorrect. She was put on modified low dose rituximab as maintenance therapy for 3 months: at 100 mg once a week for the first month, then 100 mg once a month for the next 2 months.^[21–23]^ However, she experienced partial remission after the maintenance therapy was stopped.

**Case #2:** Diagnosed with hematoma of lower limbs and presented with gingival bleeding but had no underlying disorders. At presentation, she had a prolonged aPTT of 60.9 s and FVIII inhibitor titers were 13.2 BU/mL. She was given a combination of corticosteroids (dexamethasone and prednisone) as induction therapy and 2 cycles of plasma exchange were performed on days 2 and 3. After 3 months, no FVIII inhibitors were observed and she was put on rituximab as maintenance therapy. There was complete remission, and no relapse of hemorrhage was seen during follow-up.

**Case #3:** A 49-year-old female presented with a sudden onset of severe pain in the dorsal surface of her left hand and ankle, which limited her day-to-day activities. Her past medical history included ecchymosis and subcutaneous hematoma (swelling) of both lower limbs (Fig. 1). She was previously transfused with fresh frozen plasma and received vitamin K1 for secondary coagulation dysfunction. After 1 month of therapy, her symptoms aggravated when she was diagnosed with an underlying connective tissue disease and hematoma of the lower limbs. At admission to our hospital, her Hb was 9.8 g/dL, aPTT was 80.7 s (previously 55.1 s). She showed a reduced FVIII coagulation activity of 2% (previously 6.5%), and extremely high titers of FVIII-inhibitor, 48.6 BU/mL. She was diagnosed with AHA and underwent 2 cycles of TPE and concurrently received 80 mg/d of oral methylprednisolone during the initial 4 days and 50 mg/d of oral prednisone until the 16th day, following the cessation of which there was a partial remission. She was re-admitted to the hospital. This time, she was treated with TPE twice, and rituximab 100 mg was used twice after TPE, and azathioprine was added to the hormone. However, the patient died after 11 months due to acute respiratory distress syndrome and a concomitant lung infection.

**Figure 2:**
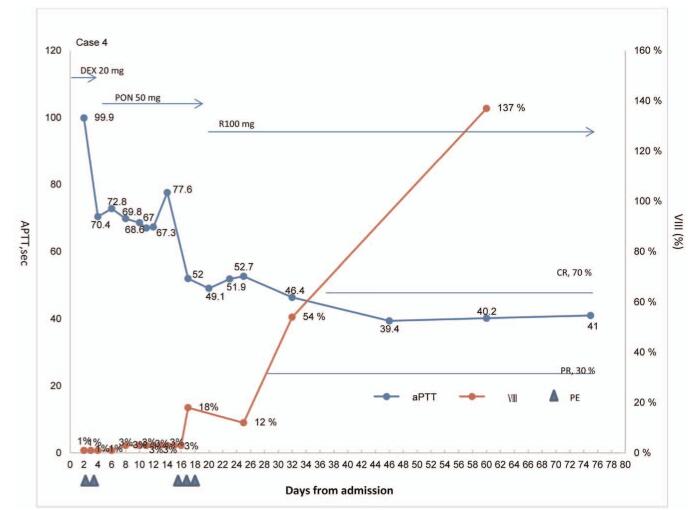
Evolution of rates of FVIII (VIII: C), activated partial thromboplastin time (aPTT) and therapies are summarized (case 4). CR = completed remission, DEX = dexamethasone, PE = plasma exchange, PON = prednisone, PR = partial remission, R = rituximab.

**Case #4**: A 33-year-old woman presented with abnormal vaginal bleeding following the delivery of a full-term baby. Upon bleeding, she was transfused with a total of 4 units of packed RBCs and 2 units of fresh frozen plasma, 6 units of cryoprecipitate, and a curettage was performed at a previous hospital. She had received oxytocin therapy and antibiotics intravenously. At admission, her Hb was 8.6 g/dL, and she showed an abnormally prolonged aPTT of 99.9 seconds, a markedly reduced FVIII activity (1%), and extremely high titers of FVIII inhibitor, 22.8 BU/mL. After a confirmed AHA diagnosis, 2 cycles of plasma exchange were performed on the 2nd and the 3rd days and in parallel, she had orally received 20 mg/d of dexamethasone for the first 4 days. Following the administration of 50 mg of oral prednisone per day from day 6 onwards. Additional cycles of plasma exchange were planned on the 16th, 17th, and 18th days due to exacerbation of vaginal bleeding on the 15th day, following which she was infused with 100 mg of rituximab per day from the 19th day onwards. Subsequently, no bleeding complications occurred, inhibitor levels decreased, and coagulation profiles significantly improved during her 25-day hospital stay. There was no relapse of hemorrhage during follow-up and complete remission was achieved. Figure 2 shows a separate chart for aPTT and FVIII activity in Case 4 alongside the days at which rituximab was received.

**Case#5:** Presented with retroperitoneal hematoma of both lower limbs and had a history of Sjogren syndrome. At presentation, he had a prolonged aPTT of 98.8 seconds. Upon confirmed AHA diagnosis, he was put on induction therapy with dexamethasone for 6 days until FVIII activity improved, and subsequently, 3 cycles of plasma exchange were performed on days 6, 9, and 11 with a parallel administration of prednisone. The patient also received rituximab on the 12th day of hospitalization. However, after the maintenance therapy was stopped, the patient experienced partial remission.

**Case #6:** An 18-year-old female presented with a 24-hour history of gastrointestinal hemorrhage (twice) persistent dizziness, weakness, and fatigue. Her past medical history included intermittent fever of unknown origin and lower back pain for the previous 3 months. Subsequently, she was diagnosed with hemolytic anemia with a progressive decline in Hb and was transfused with 2 units of packed RBCs at a previous hospital. As the cause of anemia was unknown and as her symptoms persisted at the time of admission, a bone marrow puncture was performed. Pure red cell aplasia with pre-existing SLE was confirmed. At admission, her Hb was 6.2 g/dL. She showed a markedly prolonged aPTT of 110.1 seconds, reduced FVIII activity (1%), and extremely high titers of FVIII inhibitor, 34.6 BU/mL (normal: 0–0.6 BU/mL). She was prescribed oral methylprednisolone of 60 mg/d. Subsequently, in parallel, plasma exchanges were performed from the 13th day till 16th day and were again repeated on days 18, 20, 22, and 24. Although her symptoms temporarily improved, hemorrhagic signs recurred during the treatment. Despite the administration of recombinant factor VIIa, no remission was seen. Rituximab was not administered due to abdominal infection. The patient died after a month due to a sudden abdominal infection and multiple organ failure syndromes.

## 4 Discussion

In this retrospective case series, we determined the feasibility of TPE-based combination therapy and demonstrated that TPE in combination with corticosteroids and rituximab, as adjunctive immunosuppressive agents, is an effective and reliable treatment for AHA. To the best of our knowledge, our study is the first-of-its-kind to report treatment of 6 cases who underwent TPE and received adjunctive immunosuppressants as an alternative to rFVIIa administration.

In this case series, AHA was predominantly associated with autoimmune diseases and pregnancy. Further, electronic medical records of all the patients were thoroughly reviewed to exclude patients with malignancies. Previous studies have emphasized that AHA is associated with several underlying conditions, including autoimmune disorders, infections, and malignancies. Most of the prior research acknowledged the preponderance of AHA in women in the immediate postpartum period.^[10]^ In this case series, 1 patient showed an abnormal aPTT and was diagnosed with acute postpartum hemorrhage.^[24]^ Three patients who harbored antinuclear antibodies, in addition to autoantibodies against FVIII, were diagnosed with autoimmune diseases, and 1 patient had suspected MDS, although this was later found to be incorrect. Further, a series of recent reports have indicated that the presentation of AHA in MDS is rare and complicated and that the patient may be more susceptible to a worse outcome.^[25]^ In patients without a preexisting coagulopathy, AHA was confirmed by the presence of FVIII inhibitor and a decreased FVIII activity.

Key goals in the management of AHA include control and prevention of bleeding (if present/significant), eradication of the inhibitor, and treatment of the underlying disease (if any). Furthermore, it has been well-established that successful treatment of AHA involves rapid removal of inhibitors using immunosuppressant drugs as there is a risk of severe, fatal hemorrhage during the period in which inhibitors are present.^[26–28]^ Accordingly the International guidelines recommend first-line bypassing therapy using hemostatic agents that include, administration of either recombinant FVII activated (rFVIIa) at recommended doses of 90 μg/kg, every 2–3 h until hemostasis is achieved, or bolus injection of aPCC with doses between 50 and 100 IU/kg every 8–12 hours to a maximum of 200 IU/kg/d.^[5]^ Most reports have emphasized tailored therapeutic regimens according to a patient's general condition, underlying and concomitant diseases, and prognostic factors such as FVIII <1 IU/dL, inhibitor titer >20 BU/mL, and the presence of anti-FVIII-IgA antibodies. The recommended first-line immunosuppressive therapeutic options for all patients with AHA include a monotherapy of corticosteroids, either with prednisone at doses of 1 mg/kg PO daily, or dexamethasone at doses of 40 mg PO daily for 4–7 days), combination therapy of corticosteroids and cyclophosphamide at doses of 1–2 mg/kg PO daily (or alternatively ∼5 mg/kg IV q for 3–4 weeks), or combination therapy of corticosteroids and rituximab at doses of 375 mg/m^2^ IV 4 times weekly (or, alternatively 100 mg weekly ×4). The use of rituximab was not recommended as initial monotherapy unless other immunosuppressive therapies were contraindicated.^[5]^ To avoid steroid toxicity, certain second-line drugs, such as mycophenolate mofetil, cyclosporine, and rituximab. have been used with varying successes.^[29–32]^ Although there is a bulk of research on the suitable dosage regimen and methods of administration of FVIIa for AHA, the feasibility of the combination therapy with TPE is still insufficiently explored. Therefore, this study is not only a retrospective case series but also has the potential to aid other prospective studies that evaluate the outcomes of TPE in conjunction with adjunctive immunosuppressants for the treatment of AHA.

Since there are no established/standardized treatment regimens that describe the optimal administration strategy and the optimal duration of TPE-based immunosuppressant therapies in patients with AHA, judgments were subjectively made by each attending physician. Prior research suggests that the probability of obtaining remission with steroids alone is exceptionally low.^[33]^ Of note, the investigators of the largest available registry, the European Acquired Hemophilia Registry, reported that nearly half of all the published AHA cases treated with a first-line immunosuppressant therapy that included rituximab (59%) achieved a stable CR.^[27]^ Further, patients who were treated with rituximab alone had only a 42% response rate, in contrast to those treated with rituximab and another agent, where approximately 64% stable inhibitor eradication was achieved, which is comparable to that achieved with a combination of steroid and cyclophosphamide. Given the higher probability of rituximab-based regimens for achieving a stable CR, in this study, a stable complete response, albeit a slower one, was achieved in 40% (2/5) of cases who were treated with rituximab in combination with TPE and corticosteroids (Table 2).^[34]^ The findings of this study are further corroborated by a systematic literature review of 65 patients with AHA who were treated with rituximab alone or in combination with other immunosuppressive agents, wherein more than 90% of cases, a complete or partial response was reported.

In this case series, due to cost constraints, we explored alternative approaches to eradicate FVIII inhibitors, and therefore chose TPE in addition to corticosteroids with/without rituximab. A previous report advocated that TPE played a very crucial role, resulting in the recovery of a 73-year-old male who was diagnosed with AHA and failed to respond to multiple immunosuppressive therapies including, rituximab. Consistent with previous studies, our treatment regimen involving a combination of TPE and corticosteroids with/without rituximab demonstrated a complete eradication of FVIII inhibitor and non-recurrence of bleeding symptoms. Further lending strength to our study, Rech et al showed that plasma exchange in combination with high dose corticosteroids, cyclophosphamide, and MMF, could control bleeding, normalize FVIII activity, and coagulation time in an 82-year-old male patient with AHA.^[35]^ Another report by Losos et al advocated that early initiation of TPE controlled bleeding in a 66-year-old female patient who had high titers of FVIII inhibitor, and that it played a critical role in reducing FVIII inhibitors, which resulted in a recovery of FVIII activity and hemostasis.^[36]^ Similarly, in our case series, after early initiation of TPE with fresh frozen plasma, we observed that bleeding stabilized after the first 2 treatments and completely stopped after additional treatments. FVIII inhibitors were completely eradicated after 3 months in all patients. Nonetheless, 2 patients (approximately, 33.3%) died due to the sudden onset of infections associated with preexisting underlying diseases. In line with this finding, a study by Ogawa et al reported that the mortality rate of AHA is approximately 25%, where the most frequent cause of death was infections.^[37]^

Our case series has several limitations. all cases were retrospective, small single-center study and only included 6 patients with AHA admitted to The Second Affiliated Hospital, Chongqing Medical University, which may raise confounding concerns and potential for selection bias. Further studies evaluating the benefits of this TPE-based combination therapy in larger cohorts of well-characterized patients with AHA are warranted.

In conclusion, although there is a considerable body of literature on the immunosuppressant therapies for AHA, none of them established the superiority of 1 treatment strategy over the other, it is difficult to undertake randomized controlled trials in treatments for such a rare disorder. Our results from this study indicate that TPE in combination with corticosteroids and rituximab may be an effective and promising therapy for AHA in the absence of alternatives. Our experience suggests that plasma exchange should be initiated early in AHA patients with severe bleeding and high titers of FVIII inhibitor. We advocate that a more intensive first-line treatment, including TPE may potentially achieve faster remission, without bypassing agent (rFVIIa) therapy, especially in several poor countries where that medical treatment is unaffordable.

## Author contributions

**Conceptualization:** Yun Luo.

**Data curation:** Han-Qing Zeng, Ying Zhang, Shi-Feng Lou, Jian-Chuan Deng.

**Investigation:** Shi-Feng Lou, Jian-Chuan Deng.

**Writing – original draft:** Lin-Yue Wang, Yan Shen, Yun Luo.

**Writing – review & editing:** Lin-Yue Wang, Yun Luo.
